# Impact of Microstructure on the Electrochemical Performance of Round-Shaped Pitch-Based Graphite Fibers

**DOI:** 10.3390/ma13081933

**Published:** 2020-04-20

**Authors:** Baoliu Li, Jianguang Guo, Jiajun Huang, Huitao Xu, Zhijun Dong, Xuanke Li

**Affiliations:** 1 College of Materials Science and Engineering, Hunan University, Changsha 410082, China; libl151300373@sina.com (B.L.); guojianguang@hnu.edu.cn (J.G.); HuitaoXu1995@163.com (H.X.); 2 School of Chemistry and Chemical Engineering, Wuhan University of Science and Technology, Wuhan 430080, China; jia15773113089@163.com (J.H.); dongzj72@sohu.com (Z.D.)

**Keywords:** round-shaped pitch-based graphite fibers, structure of transverse section, crystalline sizes, lithium intercalation properties, cycle performance

## Abstract

In this study, three kinds of round-shaped pitch-based graphite fiber with different microstructural features (crystallinity and carbon layer orientation) were fabricated by melt-spinning, preoxidation, carbonization and graphitization. The morphology, crystalline size and carbon layer orientation of carbon fibers from different pitch precursors and spinning rates were characterized through X-ray diffraction, scanning electron microscopy and transmission electron analyses. The correlation of the electrochemical performance and microstructure of graphite fibers as anode materials for lithium-ion batteries was investigated. The results suggest that large-diameter anisotropic graphite fibers (L-AF3000) with a radial texture of the transverse section are more favorable for lithium intercalation storage. The discharge capacity of L-AF3000 is 319.1 mAh∙g^−1^ at 0.1 *C* (current density). Nevertheless, the capacity drops to 209.9 mAh∙g^−1^ at a high current density of 1 *C*, and the capacity retention is only 82.2% over 100 cycles at 0.1 *C*. Small-diameter anisotropic graphite fibers (S-AF3000) with a spiral-shaped wrinkle texture of the transverse section possess discharge capacities of 284.1 mAh∙g^−1^ at 0.1 *C* and 260.2 mAh∙g^−1^ at a high current density of 1 *C.* Meanwhile, the best capacity retention of the fibers is 101.6% over 100 cycles at 0.1 *C*. The results suggest that the disordered carbon layers in S-AF3000 can retain the structural integrity of fibers as anode material for lithium-ion batteries and thus obtain excellent cycle stability. In addition, larger crystalline sizes of fibers correspond to higher discharge capacity, and a smaller diameter is beneficial to the fast insertion and extraction of lithium-ion in fibers.

## 1. Introduction

In recent years, driven by the ever-growing demand for “green” technologies, lithium-ion batteries (LIBs) have been extensively applied in most common portable electronic devices, such as cell phones and laptops, but also in large-scale energy storage devices, particularly electric vehicles (EVs) [1]. These energy storage systems always require higher energy densities, better cycle stability and increased rate capacity to support a longer usage time. A potentially promising way of improving power and decreasing the storage volume of these systems is to integrate rechargeable LIBs as a functional component of the mechanical structure, designing a structural battery. For example, if LIBs were used as parts of the roof of an EV, that would greatly decrease the weight and volume contributions [2]. The concept of structural batteries has previously been studied and developed with various approaches and results [3,4,5]. Among them, both mechanical and electrochemical performance goals were considered in LIB design, which is required in order to develop lightweight materials.

Carbon fibers are commonly used as structural reinforcement in composite materials because of their high tensile strength and stiffness-to-weight ratios [6,7]. Therefore, carbon fibers are considered as a potential anode candidate for structural batteries. Jacques et al. [8] reported on the most favorable carbon fiber grades for use as structural electrodes, showing that the good reversibility of the tensile property changes with the measured lithium storage capacities. On the other hand, the growing EV market also demands high battery safety to meet practical operating conditions. Large amounts of heat generated during rapid charge–discharge cycles at high current densities, such as quick acceleration, can severely affect the safety of power LIBs [9,10]. To address the above-mentioned issues, the choice and structure of carbon fibers are of key importance. Pitch-based graphite fibers, which possess higher electronic and thermal conductivities than polyacrylonitrile-based carbon fibers, have a great potential for improving the structural design and heat evacuation of LIBs [11,12]. Early in 1993, Morita et al. [13] studied the reversible lithium-ion insertion/extraction processes in pitch-based carbon fibers. Subsequently, Ohsaki et al. [14] reported the graphitized mesophase pitch-based carbon fiber as an anode material for LIBs. The results showed that the graphitized carbon fiber had a larger capacity, higher rate capability and better cycle reversibility than the powdered graphite anode. In comparison with other carbon materials, the electrochemical lithium storage behaviors of round-shaped pitch-based carbon fibers are complex and closely related to their microstructural features (crystallinity and carbon layer orientation) [15,16,17,18]. Furthermore, the microstructure of carbon fibers strongly depends on the preparation processes and carbon precursor species [19,20]. However, the correlation between the electrochemical performance and microstructure of pitch-based carbon fibers as anodes for LIBs is still not clear.

In this paper, three kinds of round-shaped pitch-based graphite fiber with different microstructural features (crystallinity and carbon layer orientation) were produced from different pitch materials and at different spinning rates. After preoxidation, carbonization and graphitization, the graphite fibers were tested as anodes for LIBs. The correlation between the microstructural features and lithium intercalation properties of the fibers was studied. Meanwhile, the effect of the carbon layers of the transverse section on the cycle performance of the fibers was also analyzed.

## 2. Experimental

### 2.1. Preparation of Round-Shaped Pitch-Based Graphite Fibers

Different molten pitch precursors were extruded through a spinneret with 20 holes of 450 mm diameter under pressurized nitrogen of 0.2 MPa at a spinning temperature of 310–330 °C. The extrudates were then drawn through a winding drum at a rotational speed of 40–80 m/min, controlled by a servo motor to form round-shaped pitch fibers. Then, as-spun isotropic and anisotropic fibers were stabilized at 280 °C and 295 °C, respectively, in an O_2_ atmosphere for 2 h at a flow rate of 200 mL/min. The obtained stabilized fibers were subsequently heat-treated to 1000 °C for 0.5 h under a N_2_ atmosphere, and then the graphitized fibers were heat-treated at 2000 °C and 3000 °C for 15 min in an Ar atmosphere.

### 2.2. Microstructure Characterization and Electrochemical Performance of Graphite Fibers

The transverse section morphologies and crystal lattice of graphite fibers were investigated using a NOVA 400 NANO field emission scanning electron microscope (SEM, Frequency Electronics, Inc., Mitchel Field, NY, USA) and a JEM 2100F high-resolution transmission electron microscope (TEM, JEOL, Tokyo, Japan), respectively. The phases present in the fibers were identified by X-ray diffraction (XRD) using a Philips X’Pert Pro MPD instrument (Amsterdam, The Netherlands) with Cu K_α_ radiation. The working voltage and current for the Cu target were 40 kV and 40 mA, respectively. The degree of graphitization (*g*) was calculated with the following Equation (1) [21]:

*g* = (0.3440 − *d*_002_)/(0.3440 − 0.3354). (1)

The crystalline parameters of graphite crystals were approximately calculated using the Scherrer Equation (2):

*L* = *Kλ*/*β*cos*θ*. (2)

Diffraction angles of {002} and {100} planes were carefully corrected by using silicon powder as an internal reference. Operation and data processing followed the standard procedure for X-ray diffraction measurements on carbon materials [22]. Electrochemical measurements were performed using 2016 coin-type cells, and lithium metal was used as the reference and counter electrodes. The working electrodes were prepared by mixing 85 wt% fibers as active materials, 5 wt% Super P (SP) as conductive additives and 10 wt% polyvinylidene fluoride (PVDF) as a binder. The electrochemical measurements were conducted in an electrolyte solution of ethylene carbonate, dimethyl carbonate and ethyl methyl carbonate (EC/DMC/EMC, volume ratio of 1:1:1) containing 1 mol∙L^−1^ LiPF_6_. The cells were tested in the potential range of 2–0.005 V vs. Li/Li^+^ at a constant current density of 0.1 *C* (37.2 mA∙g^−1^), 0.5 *C*, 1 *C* and 2 *C*. The impedances were measured after the third cycle on a CHI660D electrochemical workstation.

## 3. Results and Discussion

### 3.1. Characterization of Graphite Fibers

**Figure 1:**
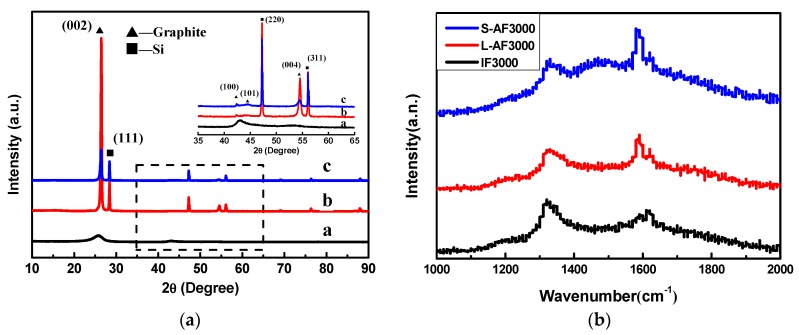
(**a**) X-ray diffraction (XRD) patterns of (a) IF3000; (b) L-AF3000 and (c) S-AF3000. (**b**) Raman spectra of three kinds of graphite fiber.

The XRD patterns of three kinds of graphite fiber are shown in Figure 1a. IF3000 (Figure 1a) showed two broad diffraction peaks, with one corresponding to the {002} plane of graphite and the other corresponding to the {100} plane. The measured FWHMs of the {002} diffraction peak were around 2.3°, and the peak indicated the turbostratic structural character of the fibers [23]. There was a sharp diffraction peak at about 2*θ* = 26.5° of L-AF3000 (Figure 1b) and S-AF3000 (Figure 1c). This peak can undoubtedly be attributed to the diffraction of {002} planes, and the presence of the sharp diffraction peak means a high degree of graphitization and a highly oriented structure of {002} planes. However, with the decrease in the diameter of anisotropic fibers, the intensity of {002} and {004} diffraction peaks decreased.

The calculations of detailed crystalline parameters are summarized in Table 1. It is clear that IF3000 showed the smallest crystallite grains and the biggest d-spacing of the {002} planes (*d*_(002)_ = 0.3483 nm). On the other hand, the average d-spacings of the {002} planes of L-AF3000 and S-AF3000 were 0.3362 and 0.3372 nm, respectively, close to the ideal graphitic d-spacings of {002} planes (*d*_(002)_ = 0.3354 nm). The *g* values of L-AF3000 and S-AF3000 were 90.6% and 79.9%, respectively. These results suggest that the larger the diameter of the fibers, the higher oriented the structure along the {002} planes of graphite; meanwhile, the *d*_002_ of anisotropic fibers increased with the decrease in fiber diameter. Furthermore, it is clear that the anisotropic graphite fibers possessed larger sizes of graphite crystals than isotropic graphite fibers. The larger sizes of graphite crystals may be beneficial to the improvement of lithium intercalation properties.

The Raman spectra of three kinds of graphite fiber are shown in Figure 1b. The symmetry of the D-line and G-line of the anisotropic fibers was better than that of the isotropic fibers. The peak shape was narrow, and the strength of the D-line was weak. In particular, L-AF3000 had a strong G-line and a weak D-line; the D/G ratio of the intensity of the two peaks was significantly lower for the IF3000 and S-AF3000 graphite fibers. These results show that the large-diameter anisotropic graphite fibers have a better crystal structure, fewer defects or amorphous carbon content and larger sizes of graphite crystals. This finding is consistent with the analysis of the microscopic crystal structure.

**Figure 2:**
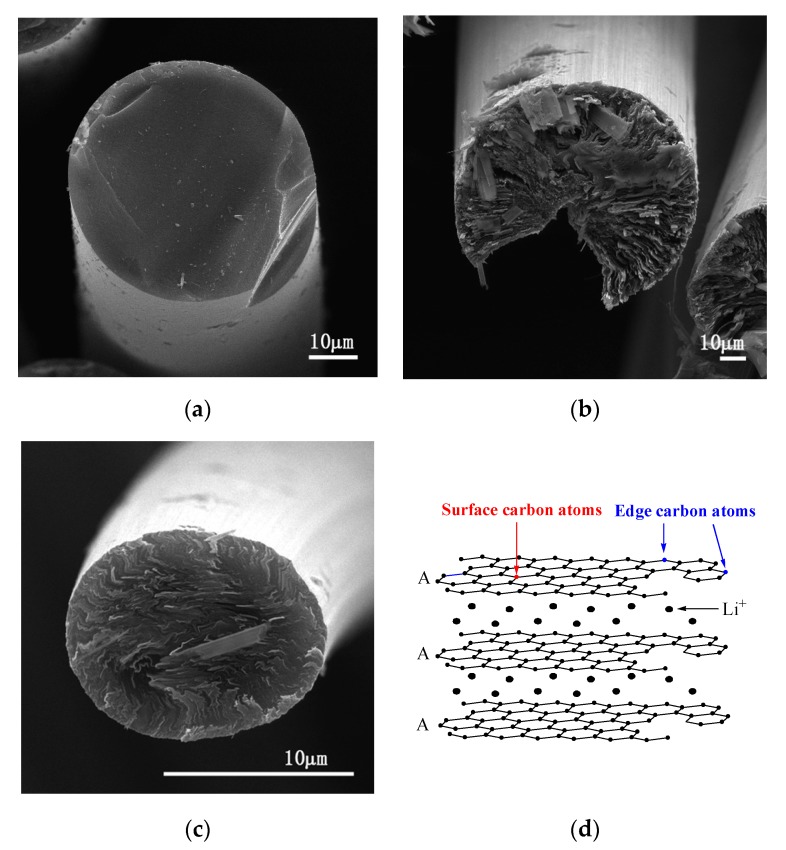
SEM images of the transverse section of (**a**) IF3000, (**b**) L-AF3000 and (**c**) S-AF3000 and (**d**) structural schematic diagram of Li–graphite intercalation compounds (GICs).

Figure 2 shows SEM images of transverse sections of the above fibers and a structural schematic diagram of Li–graphite intercalation compounds (GICs). The transverse section of IF3000 showed a complete morphology with a uniform texture structure (Figure 2a). From the SEM images, the statistical average diameter of the isotropic fibers was ca. 53 µm. As shown in Figure 2b, the average diameter of L-AF3000 was ca. 75 µm. It is clearly visible that the carbon layers of L-AF3000 were arranged along the radial direction, displaying the highly preferred orientation of carbon layers [24]. However, the carbon atoms on the surface of the graphite crystal were exposed because of a wedge-shaped site, observed in Figure 2b [25]. Figure 2c illustrates that the transverse section of S-AF3000 also had a complete morphology. However, the observed texture was radial-folded and disturbed. This means that the carbon layer orientation of S-AF3000 was subjected to stronger shear action of the spinneret and the traction force of the winding drum [26]. The increases in the spinning rate and shear action of the spinneret caused an overall decrease in the degree of order of the carbon layers. The carbon layers in the core region exhibited a spiral-shaped texture, while the carbon layers on the surface region had a wavy, wrinkled texture as well as a radial direction arrangement due to the influence of shear action of the spinneret. Contrasted with L-AF3000, the contacting electrolyte solution of S-AF3000 includes carbon atoms on the edge of a graphite crystal. The carbon atoms on the edge may be beneficial for the formation of a complete and thin solid electrolyte interface (SEI) film and an increase in the initial Coulombic efficiency [27].

**Figure 3:**
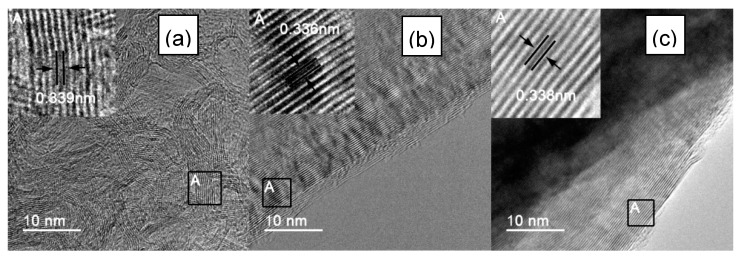
HR-TEM images of (**a**) IF3000; (**b**) L-AF3000 and (**c**) S-AF3000.

To further observe the images of the atomic lattice of IF3000, L-AF3000 and S-AF3000, HR-TEM images of these fibers are shown in Figure 3. IF3000 presented a staggered net plane of lattice fringes (Figure 3a), suggesting the disordered state of the structure of carbon layers. The measured graphene sheets had around 10–16 layers, and the part of the d-spacing of the {002} planes was calculated to be ca. 0.339 nm from the boxes marked in these figures. On the other hand, L-AF3000 presented highly oriented stacking of the {002} planes, shown in Figure 3b, and the d-spacing of the {002} planes was ca. 0.336 nm, shown in the boxes marked. Moreover, S-AF3000 (Figure 3c) also presented highly oriented stacking of the {002} planes. Nevertheless, it is easy to observe from the boxes marked in these figures that the d-spacing of the {002} planes increased to ca. 0.338 nm, and the atomic lattice became more distorted than that of L-AF3000.

### 3.2. Electrochemical Property of Graphite Fibers

**Figure 4:**
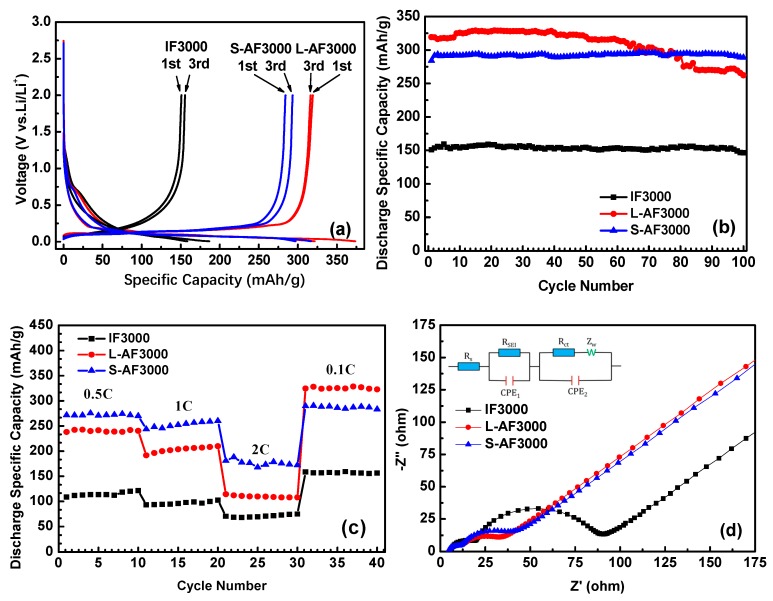
(**a**) First and third charge/discharge cycle profiles at 0.1 *C*; (**b**) cycle performance of IF3000, L-AF3000 and S-AF3000 at 0.1 *C*; (**c**) rate capacity of IF3000, L-AF3000 and S-AF3000 and (**d**) impedance spectra of IF3000, L-AF3000 and S-AF3000.

Figure 4a shows the first and third charge and discharge profiles of three kinds of fiber at 0.1 *C* charge and discharge rates. In the first-cycle curves, there is an obvious plateau around 0.75 V, which corresponds to the formation of SEI film. The plateau of S-AF3000 is shorter than those of IF3000 and L-AF3000. This result comes from the complete morphology of the transverse section shown in the SEM images (Figure 2). An increase in the contents of carbon atoms on the edge decreased the loss of lithium-ion on the formation of the SEI film in part of the contacting the electrolyte solution. Furthermore, the discharge plateau below the voltage of 0.25 V, corresponding to the formation process of Li–GICs, is in the order IF3000 < S-AF3000 < L-AF3000. The detailed data of first- and third-cycle processes are summarized in Table 2. The discharge capacity and initial Coulombic efficiency of IF3000 were only 150.9 mAh∙g^−1^ and 80.9%, respectively. In contrast, the discharge capacities of L-AF3000 and S-AF3000 were much higher, with values of 319.1 and 284.1 mAh∙g^−1^, respectively, which was due to the relatively ordered lattice fringes. Meanwhile, the initial Coulombic efficiency was 85.5% and 89.6%, respectively. This result illustrates that the lithium intercalated sites are highly dependent on both the sizes of graphite crystals and the orientation of the carbon layer. The highly preferred orientation of carbon layers and larger crystalline sizes achieved the desired enhancement in the discharge capacity of anode materials. In addition, Figure 4a also reveals Coulombic efficiencies of nearly 99% in electrodes of all graphite fibers in the third cycle (detailed data are shown in Table 2). This result suggests that Coulombic efficiencies increased rapidly and exceeded 99% in subsequent cycles. The cycle performance of overall graphite fibers is shown in Figure 4b and Table 2. It is worth noting that L-AF3000 showed the largest discharge capacities (ca. 329.1 mAh∙g^−1^) in the first 70 cycles, yet it suffered from a gradual capacity decay with increased cycle numbers. After 100 cycles, the capacity retention of L-AF3000 was only 82.2% compared to that in the first cycle. In contrast with the poor cycle stability of L-AF3000, IF3000 and S-AF3000 displayed higher capacity retentions of 97.0% and 101.6%, respectively. These results mean that the uniform or spiral-shaped texture of the transverse section dispersed the stress towards the core direction of fibers during lithium-ion intercalation processes.

Figure 4c presents the rate capability of IF3000, L-AF3000 and S-AF3000. The cells of these samples were tested at 0.5 *C* for the initial 10 cycles, and the rate was increased stepwise to as high as 2 *C* (744 mA∙g^−1^) each for 10 cycles. The capacities of S-AF3000 at 0.5, 1 and 2 *C* were 270.1, 260.2 and 172.3 mAh∙g^−1^, respectively. Moreover, when the current density returned to 0.1 *C* after 30 cycles, the fibers recovered their original capacity of 283.5 mAh∙g^−1^. At various high current densities, the capacities of S-AF3000 were markedly superior to those of its two counterparts. This result clearly suggests that the small diameter of the transverse section is favorable for the fast de-intercalation of lithium-ions from fibers. As shown in Figure 4d, the impedance spectra for overall samples are depicted as two semicircles and an inclined line. Including the initial point and the first semicircle, the high-frequency region corresponds to the ohmic resistance and Li^+^ migration resistance through the SEI film, respectively. The middle frequency corresponds to the charge-transfer resistance, which is combined in parallel with the double layer capacitance [13]. The inclined line in the lower-frequency region is related to the Warburg impedance or mass-transfer impedance, which is associated with the diffusion of lithium-ions in the active material [28]. The SEI film resistance and charge-transfer resistance of anisotropic graphite fibers (L-AF3000 and S-AF3000) were observed to be smaller than those of isotropic graphite fibers (IF3000). This result means that highly oriented structures are beneficial to the decrease in the above resistances. The charge-transfer resistance of L-AF3000 was again smaller than that of S-AF3000, which was caused by higher conductivity from the larger crystalline sizes and highly oriented carbon layers.

**Figure 5:**
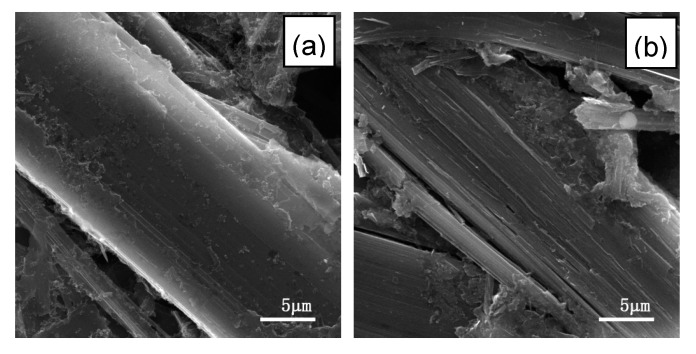
SEM images of the surface of L-AF3000 (**a**) before cycle and (**b**) after 100 cycles.

To investigate the morphology change of L-AF3000 during the cycles, the cells before and after 100 cycles were disassembled, and then the anode materials were cleaned up. The smooth and undamaged surface of virgin L-AF3000 is visible in Figure 5a. However, the fibers of L-AF3000 were divided and the structure was damaged after 100 cycles, as shown in Figure 5b. The repeated cycle processes resulted in the irreversible exfoliation of carbon layers and damage to the structure. This result may originate from the axis core of fibers subjected to more stress during the lithium-ion intercalation process.

## 4. Conclusions

A good correlation is established between capacity and crystalline sizes in round-shaped pitch-based graphite fibers. L-AF3000 possesses a higher degree of graphitization and larger crystalline sizes, whilst the discharge capacity of the fibers is 319.1 mAh∙g^−1^ at 0.1 *C*, higher than the capacities of IF3000 (150.9 mAh∙g^−1^) and S-AF3000 (284.1 mAh∙g^−1^). On the other hand, the orientation of carbon layers has a significant influence on the cycle performance. The carbon layers of L-AF3000 are arranged along the radial direction, resulting in the exfoliation of carbon layers during repeated lithium intercalation processes. The factor reduces the capacity retention, which is only 82.2% over 100 cycles. However, the capacity retention of S-AF3000 with a spiral-shaped wrinkle texture in the core of fibers is 101.6% over 100 cycles. Meanwhile, the S-AF3000 can maintain a high capacity of 260.2 mAh∙g^−1^ at 1 *C*, which is due to the structure of a small diameter of the transverse section, corresponding to 91.6% of the capacity at 0.1 *C*. In addition, IF3000 prepared by isotropic pitch has a relatively low reversible capacity and excellent capacity retention (97.0% over 100 cycles).

## Figures and Tables

**Table 1:** Crystalline parameters of different pitch-based graphite fibers.

Sample	*d*_002_ (nm)	*g* (%)	*L_a_*_(002)_ (nm)	*L_c_*_(100)_ (nm)
IF3000	0.3483	n/a ^a^	3.6	3.5
L-AF3000	0.3362	90.6	58.5	34.6
S-AF3000	0.3372	79.3	32.2	24.8

^a^ n/a: the degree of graphitization for the sample is unavailable because of the high disorder of carbon layers.

**Table 2:** Electrochemical test data of the different charge/discharge cycles at 0.1 *C* of three pitch-based graphite fibers.

Sample	1st Cycle		3rd Cycle		100th Cycle	
	Discharge Capacity (mAh∙g^−1^)	Coulombic Efficiency (%)	Discharge Capacity (mAh∙g^−1^)	Coulombic Efficiency (%)	Discharge Capacity (mAh∙g^−1^)	Capacity Retention (%)
IF3000	150.9	80.9	155.6	98.2	146.4	97.0
L-AF3000	319.1	85.5	316.4	98.4	262.3	82.2
S-AF3000	284.1	89.6	293.3	98.7	288.6	101.6
